# Discovery of Plasma Lipids as Potential Biomarkers Distinguishing Breast Cancer Patients from Healthy Controls

**DOI:** 10.3390/ijms252111559

**Published:** 2024-10-28

**Authors:** Desmond Li, Kerry Heffernan, Forrest C. Koch, David A. Peake, Dana Pascovici, Mark David, Cheka Kehelpannala, G. Bruce Mann, David Speakman, John Hurrell, Simon Preston, Fatemeh Vafaee, Amani Batarseh

**Affiliations:** 1 BCAL Diagnostics Ltd., Sydney, NSW 2000, Australia; 2 OmniOmics.ai Pty Ltd., Pagewood, NSW 2035, Australia; 3 InsightStats, Croydon Park, NSW 2133, Australia; 4 Department of Surgery, The Royal Melbourne Hospital, Parkville, VIC 3050, Australia; 5 The Peter MacCallum Cancer Centre, Sir Peter MacCallum Department of Oncology, University of Melbourne, Melbourne, VIC 3010, Australia; 6 BreastScreen Victoria, Carlton, VIC 3053, Australia; 7 School of Biotechnology and Biomolecular Sciences, University of New South Wales (UNSW), Sydney, NSW 2052, Australia

**Keywords:** lipids, breast cancer, biomarker, machine learning, liquid biopsy, cancer diagnostic

## Abstract

The development of a sensitive and specific blood test for the early detection of breast cancer is crucial to improve screening and patient outcomes. Existing methods, such as mammography, have limitations, necessitating the exploration of alternative approaches, including circulating factors. Using 598 prospectively collected blood samples, a multivariate plasma-derived lipid biomarker signature was developed that can distinguish healthy control individuals from those with breast cancer. Liquid chromatography with high-resolution and tandem mass spectrometry (LC-MS/MS) was employed to identify lipids for both extracellular vesicle-derived and plasma-derived signatures. For each dataset, we identified a signature of 20 lipids using a robust, statistically rigorous feature selection algorithm based on random forest feature importance applied to cross-validated training samples. Using an ensemble of machine learning models, the plasma 20-lipid signature generated an area under the curve (AUC) of 0.95, sensitivity of 0.91, and specificity of 0.79. The results from this study indicate that lipids extracted from plasma can be used as target analytes in the development of assays to detect the presence of early-stage breast cancer.

## 1. Introduction

Breast cancer is the most common cancer among women, excluding skin cancer, accounting for 2.3 million new cases worldwide in 2020, resulting in 685,000 deaths [1]. Treatment options for women with breast cancer have improved greatly over the last few decades, leading to a 5-year survival rate of 90–100% when diagnosed early, at stage I or II [2]. However, once the disease has metastasized (stage IV), the 5-year relative survival rate drops to 31% [3].

Early detection, diagnosis, and treatment are key to improving breast cancer outcomes. The most common early detection screening method for breast cancer is 2D mammography. However, mammograms are prone to false-positive and false-negative results that lead to undue fear/stress and missed diagnoses, respectively. Diagnosis is only made once a suspicious lesion has been identified by imaging and then confirmed by biopsy with histological examination of the tissue. Mammography also has reduced accuracy in detecting invasive cancers in women with dense breasts, which contributes to reduced mammogram sensitivity in these women [4]. These women are predominantly younger (<50 years) and more likely to have aggressive cancers [4,5]. Independent of test performance, many women do not attend mammographic screening due to fear of procedural pain or cultural barriers [6,7]. Access to facilities is also a barrier for women who live outside metropolitan areas. This underscores the importance of innovation to develop highly accurate, minimally invasive, and more easily accessible diagnostic tests for breast cancer.

A proposed solution to the challenges of breast cancer detection has been the advent of ‘liquid biopsies’. These involve the collection of biofluids (e.g., blood) that contain biological information that indicates cancer is present somewhere in the body. This information can come in the form of circulating tumor cells (CTCs), genetic material (DNA/RNA), proteins, metabolites, and lipids. Liquid biopsies rely on these specific biomarkers to enter the circulation and be present in the sample that is tested. The detection of CTCs in late-stage metastatic disease has been confirmed in multiple clinical studies [8,9,10]. Despite this, CTCs are found infrequently in metastatic disease and are even rarer in early-stage disease [11,12]. Diagnostic tests that can detect circulating tumor DNA or RNA are in development; however, as these rely on the accumulation of mutations and the existence of tumor cells or cell-free components in the circulation, these tests are better suited for the detection of later-stage disease. 

A promising source of highly concentrated tumor material found in biofluids, particularly blood, is extracellular vesicles (EVs). EVs are naturally occurring vesicles that bud from the surface of almost all cells. When released from tumor cells, they contain a small amount of DNA, RNA, and proteins encased in lipid membranes [13]. EVs have been shown to be a rich source of tumor-specific analytes for potential diagnostics methods. Furthermore, as EVs are continually generated by cells they are likely to be present not only in metastatic disease but also in early-stage disease. 

Novel biomarkers that can accurately and reliably indicate the presence of early-stage breast cancer have been difficult to identify. However, recent advances in analytical methods and new mathematical algorithms have greatly improved these efforts. Lipids are essential components of all human cells and fluids, including blood. Changes in lipid profiles are associated with the development or presence of different cancer types, including colorectal, kidney, prostate, lung, and pancreatic cancers [14,15,16,17]. Associations between changes in the abundance of certain lipids and the presence of breast cancer have previously been published for various sample types, including breast cancer cells, tissue, urine, blood serum, blood plasma, and plasma-derived EVs [18,19,20,21,22,23,24,25,26,27,28,29,30,31,32,33]. Despite various publications reporting a relationship between lipids and breast cancer, these findings are yet to be translated into a diagnostic test [34,35]. This could be due to the challenges of moving from a discovery panel of potential lipids to a panel of fully characterized lipids that can be reliably quantified using a commercially viable and analytically validated method. 

In this study, we aimed to identify a multivariate lipid biomarker signature that can distinguish healthy control individuals from those with breast cancer. We employed liquid chromatography with high-resolution and tandem mass spectrometry (LC-MS/MS) to identify both EV-derived and plasma-derived lipid signatures. An initial 23-lipid signature was identified in EVs using differential abundance and logistic regression. Machine learning approaches were also employed using plasma and EVs, where a signature of 20 lipids was identified using a feature selection algorithm based on random forest feature importance. These selected lipids served as predictor variables to train an ensemble of machine learning classifiers for jointly predicting disease status.

## 2. Results

### 2.1. Discovery of Lipids in EVs as Biomarkers of Breast Cancer

**Figure 1:**
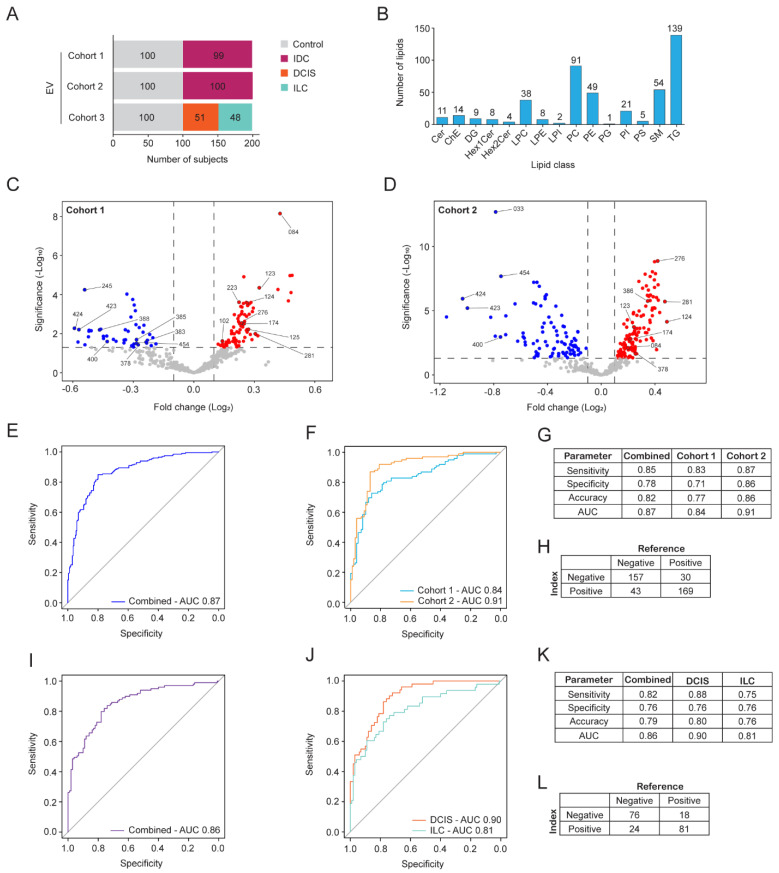
Logistic regression-based EV lipid discovery for breast cancer detection. (**A**) Overview of the sample set (n = 598) used in EV lipid discovery. EVs were enriched from plasma samples obtained from three cohorts of women with three morphologically distinct breast cancer types or healthy controls. (**B**) Number of lipid species in each lipid class that were consistently detected in cohorts 1-2. (**C**,**D**) Volcano plots of lipid profiles identified in EVs from breast cancer subjects compared to controls from (**C**) cohort 1 and (**D**) cohort 2. The fold-change in relative lipid abundance is shown. Lipid species that were significantly decreased (blue dots) or increased (red dots) in breast cancer samples are indicated, and lipids chosen for further assessment are annotated with a number (LID). (**E**,**F**) ROC curves for the internal validation using the logistic regression model for the EV23 panel to predict the presence of IDC from EVs in cohorts 1 and 2. (**E**) Cohort 1 and 2 combined. (**F**) Cohort 1 and 2 shown separately. (**G**) Model prediction outputs from (**E**,**F**). (**H**) Confusion matrix indicating model predictions from combined data in (**G**). (**I**,**J**) ROC curves for the internal validation using the logistic regression model for the EV23 panel to predict the presence of DCIS and ILC from EVs in cohort 3. (**I**) DCIS and ILC samples combined. (**J**) Predictions of DCIS and ILC shown separately. (**K**) Model prediction outputs from (**I**,**J**). (**L**) Confusion matrix indicating model predictions from combined data in (**K**). Optimized threshold in (**G**,**H**) was 0.43 and in (**K**,**L**) it was 0.41. LID, lipid identifier.

A total of 598 blood samples were obtained across three cohorts used in this study. All donors were women who were assigned female at birth. This included 300 samples from healthy donors and 298 from donors with early-stage breast cancer. The cancer morphologies included ductal carcinoma in situ (DCIS), invasive ductal carcinoma (IDC), and invasive lobular carcinoma (ILC) (Figure 1A). Donor demographics and disease characteristics were recorded, including age, body mass index, smoking status, breast cancer type, and stage (Supplementary Figure S1A–C). 

To determine if the presence of breast cancer could be detected by analyzing the lipids present in blood, EVs were isolated from the plasma fraction of blood samples. Untargeted analysis of lipids extracted from EVs was performed using high-resolution LC-MS/MS. Initial analyses were performed on samples from cohorts 1 and 2 that only contained healthy controls and individuals diagnosed with IDC. Using this discovery approach and annotation by LipidSearch software, 454 lipid species were consistently annotated across both cohorts, which could be grouped into 15 lipid classes (Figure 1B). The most frequently annotated lipid classes were triacylglycerols (TG), phosphatidylcholines (PC), lyso PCs (LPC), phosphatidylethanolamines (PE), and sphingomyelins (SM). The 454 lipid species were then evaluated for their potential to be used as biomarkers to detect breast cancer (Supplementary Table S1).

Differential abundance analysis was performed on the 454 lipid species to determine which lipids were most likely to discriminate individuals with IDC from healthy controls. A panel of 23 lipids from EVs was annotated (EV23), which corresponded to 18 distinct *m*/*z* (Figure 1C,D and Supplementary Figure S1D). These 23 lipid species were subsequently used to develop a logistic regression model with performance evaluated using leave-one-out-cross-validation (LOOCV). The model resulted in receiver operating characteristic (ROC) curves with an AUC of 0.87 when the cohorts were combined and 0.84 and 0.91 when split into separate cohorts (Figure 1E,F). Model performance exhibited an accuracy of 0.82, sensitivity of 0.85, and specificity of 0.78 when the cohorts were combined, and the threshold was set to optimize accuracy (Figure 1G). Sample level predictions are shown using a confusion matrix with this analysis (index test) compared to the pathology-confirmed sample types (reference test) (Figure 1H). The EV23 panel was then applied to cohort 3, which contains DCIS and ILC cancers. The same analyses were performed that generated an overall AUC of 0.86 and an AUC of 0.90 and 0.81 when split out into performance for DCIS and ILC, respectively. Model performance exhibited an overall accuracy of 0.79, sensitivity of 0.82, and specificity of 0.76 when the cohorts were combined, and the threshold was set to optimize accuracy (Figure 1K). Sample level predictions are shown using a confusion matrix with the index test compared to the reference test (Figure 1L). These data indicate that the EV23 panel identified in cohorts 1 and 2 using only IDC samples is generalizable to predict DCIS and ILC cancers from EVs. 

### 2.2. EV-Developed Panel Can Detect Breast Cancer in Plasma Samples

Development of a clinically meaningful blood test requires robust and reliable sample processing methods that are automatable and can be performed with high throughput. Lipid extraction from EV samples can be inherently time-consuming and less reproducible than extraction directly from plasma. A lower abundance of lipids is also observed in EVs compared to plasma lipoproteins [36], which may result in lower signal-to-noise in the detected MS signal. To determine the reproducibility of extracting and detecting the lipids in the EV23 panel, coefficients of variation (CVs) were compared in matched plasma and EVs from quality control (QC) samples. Plasma samples had greater reproducibility than matched EVs demonstrated by the CV of all EV23 lipids in plasma < 30%. In comparison, up to 18 out of 23 lipids from the EV23 panel in EVs exhibited CV > 30% (Supplementary Figure S2A).

**Figure 2:**
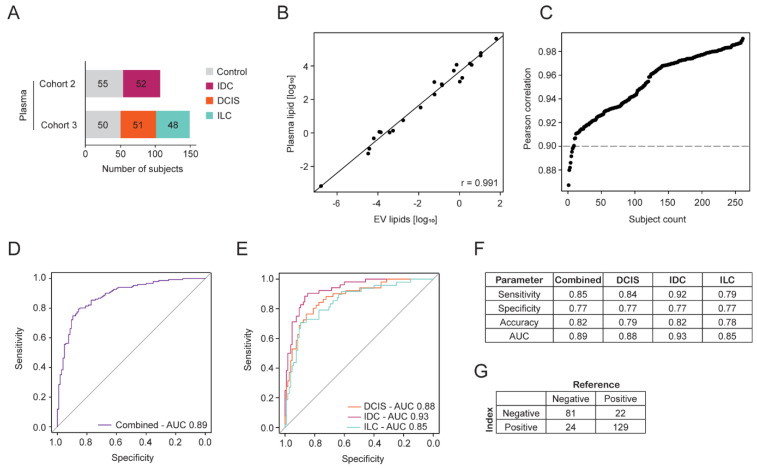
Logistic regression-based plasma lipid discovery for breast cancer detection. (**A**) Overview of the sample set (n = 256) used in plasma lipid discovery. Plasma samples are subsets of EV cohorts 2 and 3, with three morphologically distinct types of breast cancer or healthy controls. (**B**) Correlation of the lipid concentrations of the 23-lipid panel between matched EV and plasma samples was performed using the Pearson correlation method. The sample with the highest correlation coefficient (r) is shown. (**C**) The Pearson correlation coefficient for the 23-lipid panel between EVs and plasma was calculated for each of the 256 samples. The correlation coefficient for each sample is plotted and ranked by their correlation value. (**D**,**E**) ROC curves for the internal validation using the logistic regression model for the 23 lipid species panel to predict the presence of breast cancer from 256 plasma samples. (**D**) Combined predictions for all samples (n = 256). (**E**) DCIS, IDC, and ILC predictions are indicated separately. (**F**) Model prediction outputs from (**D**,**E**). (**G**) Confusion matrix indicating model predictions from combined data in (**F**). Optimized threshold in (**F**,**G**) was 0.45.

To determine if the EV23 panel could be used on plasma to predict the presence of breast cancer, a subset of 256 plasma samples from cohorts 2 and 3 were examined (Figure 2A). The reduction in donor samples in cohort 2 was performed at random and the final numbers of samples in cohorts and groups (control/IDC/ILC/DCIS) were balanced to reduce any bias in predictive model training. These cohorts were chosen as they also contained the three major breast cancer morphologies for comparison. Note that plasma contains EVs and the use of plasma in these analyses includes EV-derived and plasma-derived lipids extracted from plasma samples.

Lipids extracted from the 256 plasma samples were acquired by LC-MS/MS and the ability of the EV23 panel to predict breast cancer was evaluated. For each subject, the Pearson correlation between the EV and plasma concentrations was calculated for lipids in the EV23 panel. This indicated a strong linear relationship between the quantifications, although plasma lipids, on average, show higher concentrations (individual sample correlation, Figure 2B). The Pearson correlation (r) for each subject was plotted in rank order, resulting in 248 of the 256 subjects with a Pearson correlation > 0.9 (Figure 2C). The higher concentration of lipids in plasma supports the observed improvement in plasma CVs by reducing variability and increasing signal-to-noise ratios. Furthermore, the strong correlation of lipid changes in the two different sample sets indicates a likelihood of translating the same lipid panels developed in EVs to plasma.

Validation was performed using a logistic regression model, evaluated by LOOCV. The ability to predict the presence of breast cancers in plasma samples was similar to that observed using EV-derived lipids (Figure 1E–L and Figure 2D–G). When these data were stratified into cancer morphologies, the AUC for predicting DCIS (0.88), IDC (0.93), and ILC (0.85) were similar to one another (Figure 2E). The cancer type that was predicted most reliably was IDC, with a sensitivity of 0.92 (Figure 2F). Sample level predictions are shown using a confusion matrix with the index test compared to the reference test (Figure 2G). This indicated that despite the difference in sample input, the biomarkers identified from EVs (IDC only) were also capable of discriminating breast cancer from controls in plasma.

### 2.3. Machine Learning-Based Biomarker Panel Identification and Breast Cancer Detection from EV Samples

While differential abundance statistical analyses and logistic regression demonstrated the potential of a 23-lipid panel derived from either EV or plasma in distinguishing breast cancer patients from healthy controls, inherent limitations of the model exist. These include information leakage, failure to account for interdependent lipid effects, inability to capture nonlinear relationships, and a tendency to overfit due to LOOCV may hinder the development of a robust assay.

**Figure 3:**
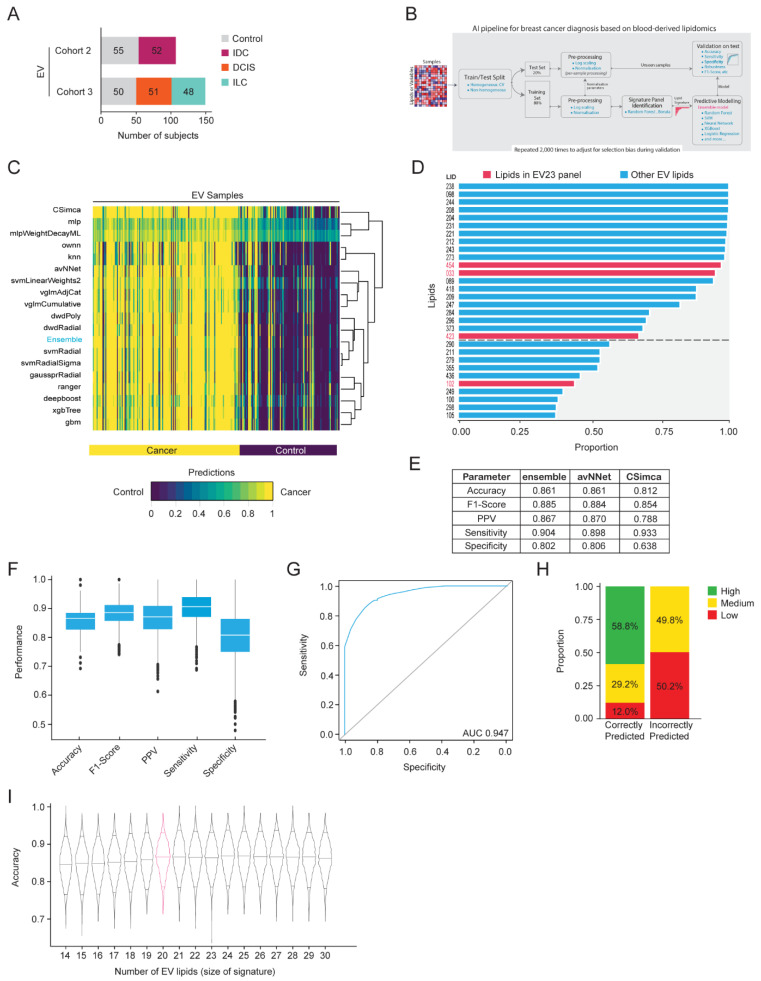
Machine learning-based EV lipid discovery for breast cancer detection. (**A**) Overview of the sample set (n = 256) used in the machine learning EV lipid discovery. EVs were enriched from plasma samples obtained from two cohorts of women with three morphologically distinct types of breast cancer or healthy controls. (**B**) A machine learning biomarker discovery pipeline was developed for signature panel identification and predictive model development. (**C**) Average prediction of each model for individual donor samples across 2000 runs. Values closer to 0 (purple) indicate a stronger prediction as control, while values closer to 1 (yellow) indicate a stronger prediction as cancer. (**D**) Lipids that are consistently selected as being important by the Boruta algorithm across all runs. The cutoff between the top 20 and the remaining 10 lipids is indicated with a dotted line. Lipids from the EV23 panel are indicated with red text and bars. (**E**–**H**) Results using the top 20 lipids from (**D**) as variables and using the (**E**) indicated models or (**F**–**H**) the ensemble model, trained using LGOCV (20% test, 80% train) and repeated 2000 times. (**E**) Test performance summary of the three models with the highest sensitivity. (**F**) Boxplots with interquartile range are indicated, representing the distribution of performance metrics. (**G**) Average ROC curve and AUC. (**H**) Certainty level of predictions. High: complete model agreement, medium: greater than 80% model agreement, low: less than 80% model agreement. Proportion (%) of high, medium, and low predictions are indicated. (**I**) Sensitivity analysis on the EV ensemble model with varying numbers of lipids. The violin plots represent the distribution of the ensemble model accuracy such that the top 14 to 30 lipids were selected based on (**D**). Horizontal lines within each violin represent the 0.05, 0.5, and 0.95 quantiles for prediction accuracy. The signature size with the best accuracy and the fewest lipids is indicated by a pink density curve. LID, lipid identifier.

To address these limitations, a rigorous machine learning pipeline was developed. The same 256 samples were used for machine learning model development based on EV-derived lipids (Figure 3A). The pipeline described in Figure 3B was used, resulting in 2000 lipid signatures and predictive modeling suites. This comprised 18 classifiers (Figure 3C) and an ensemble approach that combined the 18 classifiers, based on majority vote. Within each iteration, the hyperparameters of each classifier were optimized using a random search procedure over 50 iterations of nested leave-group-out-cross-validation (LGOCV).

The Ensemble model was the best-performing method (accuracy = 84.1 ± 4.6%, sensitivity = 89.3 ± 5.7%, specificity = 76.9 ± 9.1%), closely followed by Neural Networks Using Model Averaging [37] (accuracy = 83.4 ± 4.8%, sensitivity = 87.4 ± 6.1%, specificity = 77.9 ± 9.3%) and Distance Weighted Discrimination with a polynomial basis function [38] (accuracy = 83.3 ± 4.9%, sensitivity = 86.1 ± 6.2%, specificity = 79.4 ± 9.1%)). We selected the ensemble model for ongoing development due to its better performance and, more importantly, its capacity to generate a more generalizable predictive approach.

We investigated the proportion of lipids frequently selected as being important using the Boruta algorithm [39] across 2000 iterations of the LGOCV. Figure 3D illustrates the top 30 features (lipids) sorted by the proportion of selections (out of 2000). The top 20 EV lipids were selected as the final signature (EV20 panel) for training the ensemble model. There were four lipids identified in the top 30 that were also identified in the EV23 panel. The sum compositions for the lipid species in this EV20 panel were reported (Supplementary Figure S3A).

Hyperparameters for each classifier were chosen by the model, which obtained its median accuracy across runs where the method achieved its best rank. The median run was selected to avoid biasing models towards overly difficult or simple testing samples. The final model was trained using LGOCV (20% test, 80% train) and repeated for a total of 2000 iterations to rigorously evaluate the performance of the final model with respect to selection bias.

The average performance of the ensemble model, as well as 18 classifiers, across 2000 LGOCV iterations was reported (Supplementary Figure S3B). The three models with the highest sensitivity were the ensemble (0.904), neural networks using model averaging (avNNet, 0.898), and soft independent modeling of class analogy (CSimca, 0.933) (Figure 3E). However, the ensemble model exhibited the most stable results across iterations of different metrics (accuracy = 86.1 ± 4.4%, sensitivity = 90.4 ± 5.3%, specificity = 80.2 ± 8.7%, Figure 3F) and an AUC of 0.947 (Figure 3G). The ensemble model also represents the agreement between individual classifiers, which were used as a measure of prediction ‘certainty’. Interestingly, we observed complete agreement in classifying 58.8% of correctly predicted samples, while none of the incorrectly classified samples were predicted with high certainty (Figure 3H).

To assess the impact of reducing or increasing the number of features on the ensemble model accuracy, a sensitivity analysis was performed. Using the top 30 lipids sorted by their robustness, we reduced features one at a time and trained the model using LGOCV. The 20-lipid signature (EV20 panel) was the optimal size since increasing the signature size did not enhance accuracy and decreasing the signature size reduced the stability of model performance (Figure 3I).

### 2.4. Machine Learning-Based Biomarker Panel Identification and Breast Cancer Detection from Plasma Samples

We demonstrated that machine learning approaches can improve breast cancer predictions from EV samples compared to using simple logistic regression. Our findings indicate that lipid extraction from plasma, compared to EVs, offers greater reproducibility and scalability. Consequently, we applied our machine learning pipeline to identify a robust lipid signature and develop a predictive model capable of accurately detecting breast cancer from plasma samples.

**Figure 4:**
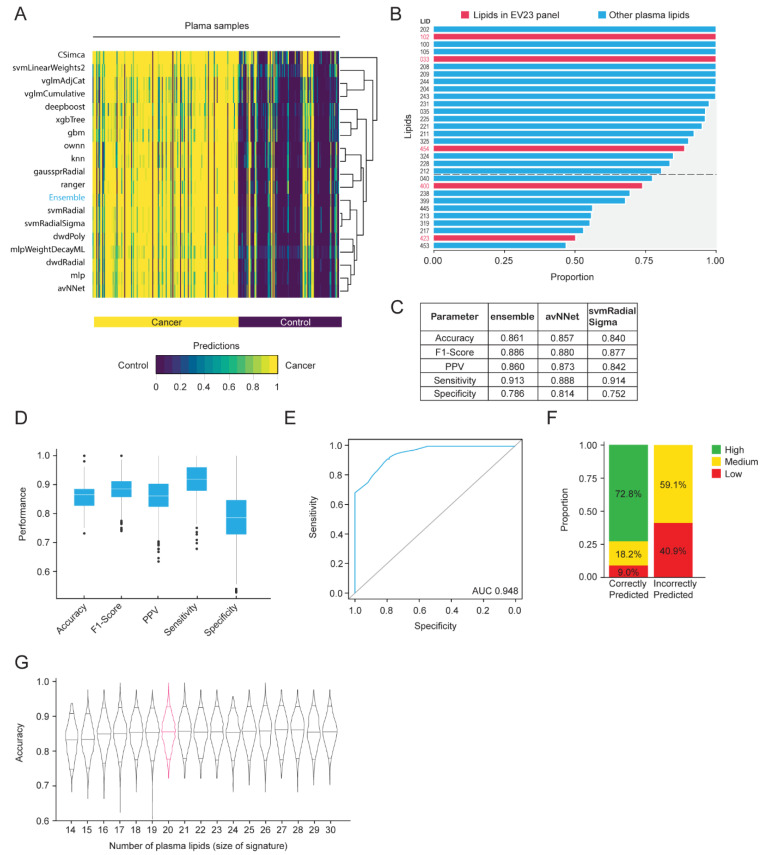
Machine learning-based plasma lipid discovery for breast cancer detection. Matched plasma from the same sample set as Figure 2A (n = 256) was used, and the same machine learning biomarker discovery pipeline as Figure 3B was used for plasma signature panel identification and predictive model development. (**A**) Average prediction of each model for individual samples across 2000 runs of LGOCV. (**B**) Lipids that were consistently selected as being important by the Boruta algorithm across all runs. The cutoff between the top 20 and the remaining 10 lipids is indicated with a dotted line. Lipids from the EV23 panel are indicated with red text and bars. (**C**–**F**) Results using the top 20 lipids from (**B**) as variables and using the (**C**) indicated models or (**D**–**F**) the ensemble model, trained using LGOCV (20% test, 80% train) and repeated 2000 times. (**C**) Test performance summary of the three models with the highest sensitivity. (**D**) Boxplots with interquartile range are indicated, representing the distribution of performance metrics. (**E**) Average ROC curve and AUC. (**F**) Certainty level of predictions. High: complete model agreement, medium: greater than 80% model agreement, low: less than 80% model agreement. Proportion (%) of high, medium, and low predictions are indicated. (**G**) Sensitivity analysis on the plasma ensemble model with varying numbers of lipids. The violin plots represent the distribution of the ensemble model accuracy such that the top 14 to 30 lipids were selected based on (**B**). Horizontal lines within each violin represent the 0.05, 0.5, and 0.95 quantiles for prediction accuracy. The signature size with the best accuracy and the fewest lipids is indicated by a pink density curve. LID, lipid identifier.

The same machine learning discovery pipeline used for EVs was implemented for the 256 matched plasma samples and the average predictions from the 19 models were generated (Figure 4A). The Ensemble model (majority vote) was the best performing method (accuracy = 84.8 ± 4.6%, sensitivity = 89.6 ± 5.7%, specificity = 78.2 ± 9.0%), closely followed by Distance Weighted Discrimination with a Radial basis function [38] (accuracy = 84.6 ± 4.8%, sensitivity = 88.8 ± 5.8%, specificity = 78.7 ± 9.1%) and Neural Networks Using Model Averaging [37] (accuracy = 84.4 ± 4.8%, sensitivity = 87.4 ± 6.3%, specificity = 80.2 ± 8.9%).

We identified the top 30 lipids that were important for predicting cancer and control samples using the Boruta algorithm across 2000 iterations of LGOCV (Figure 4B). The top 20 plasma lipids were selected as the final signature (P20 panel) for training the ensemble model. There were five lipids identified in the top 30 that were also identified in the EV23 panel. The sum compositions for the lipid species in this P20 panel were reported (Supplementary Figure S4A).

The same approach that was used to generate the EV final ensemble model was applied to construct the plasma final ensemble model, using LGOCV (20% test, 80% train). The average performance of the ensemble model, as well as 18 classifiers, across 2000 LGOCV iterations was reported (Supplementary Figure S4B). The three models with the highest sensitivity were the ensemble (0.913), neural networks using model averaging (avNNet, 0.888), and support vector machine with radial sigma kernel (svmRadial Sigma, 0.914) (Figure 3C). However, the ensemble model exhibited the most stable results across iterations of different metrics (accuracy = 86.1 ± 4.5%, sensitivity = 91.4 ± 5.4%, specificity = 78.7 ± 8.6%, Figure 4D) and had an AUC of 0.948 (Figure 4E). We also observed complete agreement in classifying 72.8% of correctly predicted samples, while none of the incorrectly classified samples was predicted with high certainty (Figure 4F).

A sensitivity analysis was also conducted using the top 30 plasma lipids, ranked by their robustness. We iteratively reduced the number of features, training the model with each subset using LGOCV, and identified the 20-lipid signature (P20 panel) as the optimal size (Figure 4G).

### 2.5. Comparison of Lipid Signatures and Predictive Performance from EV and Plasma Models

**Figure 5:**
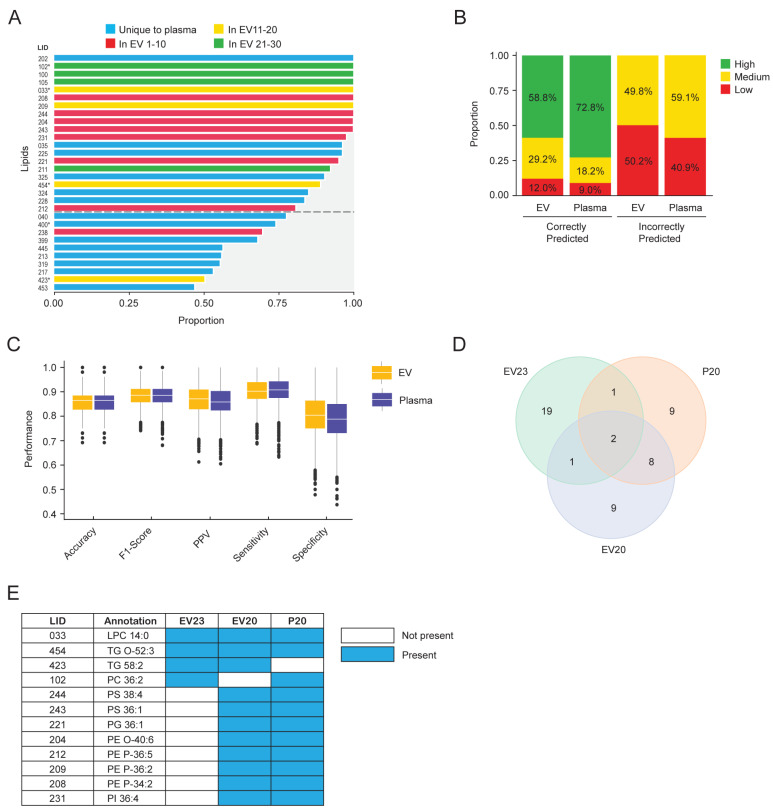
Comparison of Plasma- and EV-derived lipid signatures to predict breast cancer. (**A**) Comparison of the lipids that were consistently selected as being important in both plasma and EVs using the machine learning discovery pipeline. Lipids that were consistently selected as being important by the Boruta algorithm across all plasma runs are shown. Blue bars indicate lipids that were unique to the plasma analysis. Red, yellow, and green bars indicate lipids that were also identified in the top 30 lipids in the EV analysis. Lipids identified in the EV23 panel are indicated with an asterisk (*) next to the LID. The cutoff between the top 20 and the remaining 10 lipids is indicated with a dotted line. (**B**,**C**) Comparison of the results using the top 20 lipids from plasma and EVs and using the ensemble model. (**B**) Certainty level of predictions on correctly classified and incorrectly classified samples. High: complete model agreement, medium: greater than 80% model agreement, low: less than 80% model agreement. Proportion (%) of high, medium, and low predictions are indicated. (**C**) Boxplots and the interquartile range representing the distribution of the indicated performance metrics. (**D**) Venn diagram indicating how the EV 23 panel (EV23), Boruta plasma 20-lipid panel (P20), and Boruta EV 20-lipid panel (EV20) overlap. (**E**) LID and sum composition annotation of lipid species that were found in the overlapping regions of the Venn diagram in (**D**). Lipid species that have been described in association with breast cancer in the literature are indicated. LID, lipid identifier.

We compared each of the methods used in this study. The focus of the evaluation was sensitivity since this indicates how often the predictive model can correctly identify a sample from an individual with cancer. Compared to the EV23 panel and logistic regression model developed using differential abundances, the machine learning and Boruta feature selection method used with the matched EV and plasma samples gave higher sensitivity (0.82 and 0.85 compared to 0.904 ± 0.053 and 0.914 ± 0.054, respectively). Accordingly, the lipid panels identified to best predict control and cancer samples were compared. In the P20 panel, 6 lipids were unique to plasma, and the other 14 were identified in the top 30 lipids from EVs (Figure 5A). There were also eight lipids that were unique to the EV20 panel (Supplementary Figure S5A). The P20 panel generated results with greater certainty than the EV20 panel, with 72.8% compared to 58.8% in complete agreement, respectively (Figure 5B). Ensemble model performance was similar between EV (EV20 panel) and plasma (P20 panel) (Figure 5C). When comparing the lipids that were identified using the different methods and sample types, only two lipids were identified in all three signatures (Figure 5D and Supplementary Figure S5B). LPC 14:0 and TG O-52:3 were the lipid species annotated in all three panels, while 10 other lipid species were identified in two out of the three panels (Figure 5E). Of those, eight were identified in both EV20 and P20 panels, which included lipid species from the PS (2), PG (1), PE (4) and PI (1) lipid classes. Five of the twelve lipids have not been described previously. The remaining seven lipid annotations have been associated with breast cancer, including LPC 14:0 [28,33,40,41], PC 36:2 [23,27,33,42], PS 38:4 [33], PS 36:1 [33], PE O-40:6 [33], PE P-34:2 [30] and PI 36:4 [43].

## 3. Discussion

The overarching goal of breast cancer screening is to detect tumors as early as possible because this provides patients with the best chance of survival. Tests based on biomarkers in the blood are routinely used in cancer care but their use in diagnostics is less common. A blood-based biomarker assay for the detection of breast cancer presents an attractive and convenient method for screening. Current approaches focus on the analysis of genetic mutations, circulating tumor cells or nucleotides, or proteins for the detection of cancer. So far, the development and clinical utility of a lipidomics-based cancer detection assay has not been demonstrated. In this study, we used LC-MS/MS to discover potential lipid biomarkers that can distinguish healthy controls and individuals with early-stage breast cancer using blood plasma samples.

Linear regression and more complicated machine learning approaches both indicated the applicability of lipidomics to detect breast cancer. We were able to detect the three major morphological sub-types of breast cancer with high accuracy, sensitivity, and specificity. Similar test performances were also observed between EV- and plasma-derived lipid panels in this study. This could, in part, be due to the process itself of extracting lipids from plasma, as plasma contains EVs. Given we were able to predict the presence of breast cancer using plasma samples with a sensitivity of 0.85–0.91 across all models, it is plausible that we were detecting changes in lipid profiles based on alterations to lipid metabolism by breast cancer cells or stromal cells in the tumor microenvironment. Based on the previous literature, it is likely that the changes in lipid concentrations are a result of complex signaling pathways, which lead to the accumulation and consumption of lipids that favor tumor cell proliferation and migration [44,45]. In order to draw more specific conclusions on how plasma lipid profiles are altered in breast cancer, further research is required. As this was an untargeted discovery study using LC-MS/MS, we did not identify the full molecular structure of specific lipids, which would be required to accurately interrogate and define the signaling pathways involved. Instead, we have reported the sum composition, or annotation, for each of the lipid species we recorded. Identification of lipids using mass spectrometry requires authentic reference standards to be used for each lipid, which we intend to use in future studies with a refined panel of lipids.

The sum composition of lipids is commonly reported in the literature, which enables the comparison of breast cancer biomarker studies using these annotations. Five of the twelve lipid species we annotated across multiple panels have not been described previously as biomarkers for breast cancer. Of these five, only TG O-52:3 was important in all three panels we identified in this study. This may indicate that a lipid with this annotation could be used as a reliable biomarker for breast cancer detection. Besides this study, TG O-52:3 has only been associated with pediatric non-alcoholic fatty liver disease [46]. The remaining four lipid species have been associated with stroke recurrence after transient ischemic attack (TG 58:2) [47], MEGDEL syndrome (PG 36:1) [48], pancreatic cancer (PE P-36:5) [49], and cervical cancer (PE P-34:2) [50].

The P20 lipid panel had the best performance in this study and was developed in plasma using an ensemble of machine learning models. This approach involved identifying multivariate features using multiple subsampling of training data, constructing nonlinear predictive models, and evaluating performance through multiple iterations of LGOCV. The machine learning pipeline was designed to reduce information leakage, account for complex interactions among lipids, and reduce the risk of overfitting by running multiple iterations utilizing only 80% of the samples, simulating different sampling of the population, thereby enhancing the reliability and generalizability of the model. This was a robust model, however, a limitation of the generalizability of this model is that the plasma samples came from a relatively localized Caucasian population in Europe. Plasma lipid expression is epigenetically regulated [51] and, therefore, it is possible that the signatures developed in this discovery study are less generalizable across geographic and ethnically diverse populations. As such, future studies will include geographic and genetic diversification of the training and testing samples to improve the generalizability of the lipid signature to detect breast cancer.

LC-MS/MS was chosen as the base technology for the potential development of a breast cancer detection assay in this study because it is well suited for both the discovery of lipid biomarkers and for the performance of a diagnostic test. The strength of applying an LC-MS/MS methodology is that it enables the separation and identification of specific lipid molecules and their isomers, which cannot be achieved by conventional chemical or enzymatic assays as they lack isomeric specificity. Meanwhile, LC-MS/MS can also be used for the commercial development of scalable diagnostic tests as it is routinely used in clinical laboratories globally [52].

The current study used a discovery LC-MS/MS method that enabled annotation of lipids with relative differences in abundance between control and cancer samples. Although the discovery of individual biomarkers is an important step toward the development of a scalable assay, the ability to accurately and reproducibly quantify these lipids is essential. Accurate quantification in lipidomics requires a robust method to control the variability of lipid extraction, ionization efficiency, and systemic drift in the mass spectrometer. The addition of stable isotope labeled authentic internal standards to samples prior to processing allows for compensation of these sources of variability [53]. Future studies will focus on the development of the appropriate standards and methods to reliably quantify lipids to support the development of a clinically meaningful assay for the detection of early-stage breast cancer.

## 4. Material and Methods

### 4.1. Blood Samples

Fasted blood samples were prospectively collected throughout 2018–2021 from female participants at multiple sites in Eastern Europe. The presence of early-stage breast cancers (stage 0-II) in treatment naïve patients was confirmed by tissue biopsy. Healthy controls had not been previously diagnosed with breast cancer. The collected blood samples were kept at 4 °C and processed into plasma. Samples were stored at −80 °C until analysis. Cohorts included healthy controls and individuals with early-stage IDC, ILC, or DCIS. QC plasma was obtained from the Australian Red Cross LifeBlood (Alexandria, NSW, Australia).

### 4.2. EV Isolation

Isolation of EVs from plasma was performed by ultracentrifugation. Briefly, plasma samples were thawed at 4 °C and centrifuged at 3000× *g*, 15 min, 4 °C to pellet cellular debris. 900 µL of supernatant was diluted with 400 µL Dulbecco′s Phosphate Buffered Saline (DPBS) and centrifuged at 100,000× *g*, 18 h, 4 °C to pellet EVs. The supernatant was discarded, and 1400 µL DPBS was added to rinse the pellet. The pellet was centrifuged at 100,000× *g*, 1.5 h, 4 °C, and the supernatant was discarded.

### 4.3. Chemicals and Standards

Solvents for analysis including acetonitrile, methanol, 2-propanol (IPA), and water Optima™ LC/MS grade were purchased by Thermo Fisher Scientific (Waltham, MA, USA). Ammonium formate, LiChropur™, and methyl tert-butyl ether (MTBE) were purchased from Sigma-Aldrich (St. Louis, MO, USA). Butylated hydroxy toluene (BHT) and formic acid Optima™ LC/MS grade were purchased from Thermo Fisher Scientific. Medronic acid was purchased from Merck (Darmstadt, Germany). SPLASH^®^ LIPIDOMIX^®^ Mass Spec Standard, a labeled internal standard (ISTD) mixture used for quantitative analysis, and other lipid standards were purchased from Avanti Polar Lipids (Alabaster, AL, USA). NIST 1950 metabolites in human plasma, a standard reference material used for QC, were purchased from Sigma-Aldrich.

### 4.4. Sample Preparation

Lipid extraction was performed using the Matyash method [54], a 2-phase lipid extraction using MTBE, and the entire EV pellet or a 10 µL aliquot of plasma. To each sample 250 µL of MeOH with 0.01% BHT (*w*/*v*) and 50 µL of ISTD mixture containing 20 µM Cer d18:1/17:0, 20 µM Hex1Cer d18:1/12:0, 20 µM Hex2Cer d18:1/12:0, 4 µM S1P d17:1, 20 µM SPH d17:1, 4 µM SHexCer d18:1/12:0 and SPLASH^TM^ Lipidomix (20 µM PC 15:0/18:1-D_7_, 0.75 µM PE 15:0/18:1-D_7_, 0.5 µM PS 15:0/18:1-D_7_, 3.5 µM PG 15:0/18:1-D_7_, 1.0 µM PI 15:0/18:1-D_7_, 1.0 µM PA 15:0/18:1-D_7_, 4.5 µM LPC 18:1-D_7_, 1.0 µM LPE 18:1-D_7_, 50 µM ChE 18:1-D_7_, 0.5 µM MG 18:1-D_7_, 1.5 µM DG 15:0/18:1-D_7_, 6.5 µM TG 15:0/18:1-D_7_/15:0, 4.0 µM SM d18:1/18:1-D_9_, and 25.0 µM Chol-D_7_) was added. Samples were sonicated in a water bath for 10 min at 4 °C. MTBE, 1 mL was added, and the samples were sonicated for 30 min at 4 °C. A total of 250 µL of water was added, and the samples were placed on a rotary mixer for 30 min at 4 °C. Samples were then centrifuged at 16,100× *g* for 10 min at 4 °C to induce phase separation. The top organic layer was collected and evaporated to dryness in a vacuum centrifuge. Samples were reconstituted in 100 µL 2:1 IPA/methanol prior to injection into the LC-MS/MS system.

### 4.5. Reversed-Phase Chromatography

Lipid extracts were separated using a Vanquish™ Duo Ultra High-Performance Liquid Chromatography system (Thermo Scientific, Waltham, MA, USA) and an Acclaim™ C30 UHPLC column 150 × 2.1 mm, 3 μm (Thermo Scientific, Waltham, MA, USA). The chromatographic mobile phases A (60% acetonitrile, 40% water containing 10 mM ammonium formate, 0.1% formic acid, and 5 μM medronic acid) and B (90% IPA, 10% acetonitrile containing 10 mM ammonium formate, 0.1% formic acid) were used for both positive and negative ionization. The solvent gradient applied was 30% B—0 min, 43% B—2.0 min, 55% B—2.1 min, 65% B—9.0 min, 85% B—14.0 min, 100% B—16.0 min, 100% B—18.0 min, 100% B, 0.60 mL/min—18.1 min, 100% B, 0.60 mL/min—20.0 min, 30% B, and 0.40 mL/min—20.1 to 25.0 min. The initial flow rate was 0.40 mL/min, and the injection volume was 5 µL for plasma and 10 µL for EV samples.

### 4.6. LC-MS/MS Analysis

LC-MS/MS analysis of plasma lipid extracts was performed using an Orbitrap Fusion or Orbitrap Fusion Lumos Tribrid mass spectrometer (Thermo Scientific, San Jose, CA, USA) operated with an Optamax HESI source and high-flow needle installed. Profiling was conducted at 120,000 MS resolution. An LC-MS^n^ method was employed to annotate potential lipid biomarkers using data-dependent LC-MS^2^ to trigger the acquisition of lipid-specific information using MS^2^/MS^3^ (ion trap collision-induced dissociation scans) for more confident annotation of lipid mixtures [55]. If the positive ion data-dependent MS^2^ scan gave an *m*/*z* 184.0733 product ion (PC), 141.0193 neutral loss (PE), or 185.0089 neutral loss (PS), then a single CID MS^2^ experiment was conducted to further characterize the phospholipid. During the time from 1625 min, if fatty acid neutral losses were detected, then a single CID MS^2^ experiment was conducted, and the top 5 fatty acid neutral losses were selected for CID MS^3^ scans to characterize cholesterol ester, diacylglycerol, and triacylglycerol lipids. 

### 4.7. Lipid Annotation

Dedicated software is required for automated lipid annotation combining complex positive and negative ion LC-MS/MS data [56,57]. LipidSearch 4.2 (Thermo Scientific, San Jose, CA, USA) software provided automated annotation of lipids from LC-MS^n^ data and relative quantitation using stable isotope labeled internal standards [58,59]. Each lipid species annotated was assigned an arbitrary lipid identifier (LID) number.

### 4.8. Quantitative Analysis and QC

Peak integration of the high-resolution MS data was performed for quantitative analysis using Skyline v21.1.0.146 software (MacCoss Lab Software, University of Washington, Seattle, WA, USA). Relative quantitation was performed by calculating the ratio of each lipid to the ISTD from the corresponding lipid class. The reproducibility of the methodology was assessed by analyzing QC samples within each analytical batch (a minimum of 5 replicates per cohort). Lipid concentrations were then log-transformed to stabilize the variance.

### 4.9. Differential Abundance Statistical Analyses

Batch normalization (median and internal reference scaling normalization [60]) was performed to align batches within cohorts and to align cohort 1 and cohort 2. EV model development was performed by filtering annotated lipid species based on their ability to distinguish between control and cancer in cohorts 1 and 2 using ANOVA and mixed effect models (nlme version 3.1 R package implementation). The filtered set of markers was ranked based on differential abundance between control and cancer samples, selecting the top markers based on statistical (fdr adjusted *p* < 0.05) and fold change criteria (FC > 1.2). If isomeric species were present for a selected lipid, then the isomers were also selected. The filtered sets were further restricted to the final lipids according to mass to charge (*m*/*z*) by stepwise regression. Differentially abundant EV lipids in breast cancer patients compared to healthy controls (combined cohort 1 and cohort 2) were annotated and used as variables in a logistic regression model. This model was trained and validated using LOOCV. A prediction score was assigned to each sample between 0 and 1 with a threshold set for each dataset to optimize for accuracy: scores > threshold were predicted as cancer, and scores < threshold were predicted as controls. All statistical analyses were conducted in R using the basic stats (R version 4.2.2), caret version 6.0, and e1071 version 1.7 packages.

### 4.10. Machine Learning-Based Biomarker Discovery and Predictive Modelling

A machine learning-based pipeline was developed for signature panel identification and predictive model development. The process involved 2000 iterations of LGOCV with an 80% train and 20% test split to mitigate selection bias and enhance confidence in the generalizability of the results. Within each iteration, signature panel identification was conducted, 18 classification models were trained, and their performance was evaluated on the held-out test data, as detailed below.

#### 4.10.1. Signature Panel Identification

Within each iteration of LGOCV, a subset of lipids was selected for use in the predictive modeling using the 80% training set. These lipids were selected by Boruta, a robust, statistically rigorous feature selection algorithm based on random forest feature importance [39]. A *p*-value cut-off of 0.01 (Bonferroni adjusted) was used to identify consistently important features over 100 iterations with 500 trees per random forest.

#### 4.10.2. Predictive Modelling

A diverse range of 18 predictive classification models provided by the ‘caret’ package was identified for use in training an ensemble model [61]. Within each iteration of LGOCV, the predictive models were provided with the features selected by Boruta for the 80% training set. Hyperparameter selection was performed for each model using a random search with a tuning length of 10 over 50 iterations of a nested LGOCV (splitting the training set further into 80% sub-train and 20% sub-test). Upon selecting the ideal set of hyperparameters, the model was refit using the entire training set.

#### 4.10.3. Model Validation

The optimized models were subsequently validated on the held-out 20% test set. Individual model predictions were obtained for each test sample. A prediction score was assigned to each sample between 0 and 1 with a threshold of 0.5: scores > 0.5 were predicted as cancer, and scores < 0.5 were predicted as controls. In the case of the ensemble model, predictions were obtained according to a majority vote across each model, with ties being predicted as cancer.

#### 4.10.4. Final Ensemble Model

Informed by analysis of model validation results, a biomarker panel was specified, and the ideal hyperparameters for each algorithm were determined. The LGOCV procedure was repeated, this time keeping the feature set and algorithm hyperparameters constant. Models were repeatedly trained on a random 80% data split and validated on the remaining 20%.

## Data Availability

Data is contained within the article or Supplementary Material.

## Supplementary Materials

The following supporting information can be downloaded at: https://www.mdpi.com/article/10.3390/ijms252111559/s1.

## References

[1] Arnold M., Morgan E., Rumgay H., Mafra A., Singh D., Laversanne M., Vignat J., Gralow J.R., Cardoso F., Siesling S. (2022). Current and future burden of breast cancer: Global statistics for 2020 and 2040. Breast.

[2] Weiss A., Chavez-Mac Gregor M., Lichtensztajn D.Y., Yi M., Tadros A., Hortobagyi G.N., Giordano S.H., Hunt K.K., Mittendorf E.A. (2017). Validation Study of the American Joint Committee on Cancer Eighth Edition Prognostic Stage Compared with the Anatomic Stage in Breast Cancer. JAMA Oncol..

[3] Surveillance Research Program, National Cancer Institute SEER*Explorer. Breast Cancer—SEER 5-Year Relative Survival Rates, 2013–2019, by Stage at Diagnosis, Female, All Races/Ethnicities, All Ages. https://seer.cancer.gov/explorer/.

[4] Wanders J.O.P., Holland K., Veldhuis W.B., Mann R.M., Pijnappel R.M., Peeters P.H.M., van Gils C.H., Karssemeijer N. (2017). Volumetric breast density affects performance of digital screening mammography. Breast Cancer Res. Treat..

[5] Weigel S., Heindel W., Heidrich J., Hense H.-W., Heidinger O. (2017). Digital mammography screening: Sensitivity of the programme dependent on breast density. Eur. Radiol..

[6] Al-Zalabani A.H., Alharbi K.D., Fallatah N.I., Alqabshawi R.I., Al-Zalabani A.A., Alghamdi S.M. (2018). Breast Cancer Knowledge and Screening Practice and Barriers among Women in Madinah, Saudi Arabia. J. Cancer Educ..

[7] Tsapatsaris A., Babagbemi K., Reichman M.B. (2022). Barriers to breast cancer screening are worsened amidst COVID-19 pandemic: A review. Clin. Imaging.

[8] Lucci A., Hall C.S., Lodhi A.K., Bhattacharyya A., Anderson A.E., Xiao L., Bedrosian I., Kuerer H.M., Krishnamurthy S. (2012). Circulating tumour cells in non-metastatic breast cancer: A prospective study. Lancet Oncol..

[9] Rack B., Schindlbeck C., Jückstock J., Andergassen U., Hepp P., Zwingers T., Friedl T.W.P., Lorenz R., Tesch H., Fasching P.A. (2014). Circulating Tumor Cells Predict Survival in Early Average-to-High Risk Breast Cancer Patients. JNCI J. Natl. Cancer Inst..

[10] Dirix L., Buys A., Oeyen S., Peeters D., Liègeois V., Prové A., Rondas D., Vervoort L., Mariën V., Van Laere S. (2022). Circulating tumor cell detection: A prospective comparison between CellSearch^®^ and RareCyte^®^ platforms in patients with progressive metastatic breast cancer. Breast Cancer Res. Treat..

[11] Thery L., Meddis A., Cabel L., Proudhon C., Latouche A., Pierga J.-Y., Bidard F.-C. (2019). Circulating Tumor Cells in Early Breast Cancer. JNCI Cancer Spectr..

[12] Matikas A., Kotsakis A., Apostolaki S., Politaki H., Perraki M., Kalbakis K., Nikolaou M., Economopoulou P., Hatzidaki D., Georgoulias V. (2022). Detection of circulating tumour cells before and following adjuvant chemotherapy and long-term prognosis of early breast cancer. Br. J. Cancer.

[13] Kalluri R., LeBleu V.S. (2020). The biology, function, and biomedical applications of exosomes. Science.

[14] Wolrab D., Jirásko R., Cífková E., Höring M., Mei D., Chocholoušková M., Peterka O., Idkowiak J., Hrnčiarová T., Kuchař L. (2022). Lipidomic profiling of human serum enables detection of pancreatic cancer. Nat. Commun..

[15] Wolrab D., Jirásko R., Peterka O., Idkowiak J., Chocholoušková M., Vaňková Z., Hořejší K., Brabcová I., Vrána D., Študentová H. (2021). Plasma lipidomic profiles of kidney, breast and prostate cancer patients differ from healthy controls. Sci. Rep..

[16] Wang G., Qiu M., Xing X., Zhou J., Yao H., Li M., Yin R., Hou Y., Li Y., Pan S. (2022). Lung cancer scRNA-seq and lipidomics reveal aberrant lipid metabolism for early-stage diagnosis. Sci. Transl. Med..

[17] Kurabe N., Hayasaka T., Ogawa M., Masaki N., Ide Y., Waki M., Nakamura T., Kurachi K., Kahyo T., Shinmura K. (2013). Accumulated phosphatidylcholine (16:0/16:1) in human colorectal cancer; possible involvement of LPCAT4. Cancer Sci..

[18] Blücher C., Zilberfain C., Venus T., Spindler N., Dietrich A., Burkhardt R., Stadler S.C., Estrela-Lopis I. (2019). Single cell study of adipose tissue mediated lipid droplet formation and biochemical alterations in breast cancer cells. Analyst.

[19] Min H.K., Kong G., Moon M.H. (2010). Quantitative analysis of urinary phospholipids found in patients with breast cancer by nanoflow liquid chromatography–tandem mass spectrometry: II. Negative ion mode analysis of four phospholipid classes. Anal. Bioanal. Chem..

[20] Hammad L.A., Wu G., Saleh M.M., Klouckova I., Dobrolecki L.E., Hickey R.J., Schnaper L., Novotny M.V., Mechref Y. (2009). Elevated levels of hydroxylated phosphocholine lipids in the blood serum of breast cancer patients. Rapid Commun. Mass Spectrom..

[21] Shah F.D., Shukla S.N., Shah P.M., Patel HR H., Patel P.S. (2008). Significance of Alterations in Plasma Lipid Profile Levels in Breast Cancer. Integr. Cancer Ther..

[22] Chen X., Chen H., Dai M., Ai J., Li Y., Mahon B., Dai S., Deng Y. (2016). Plasma lipidomics profiling identified lipid biomarkers in distinguishing early-stage breast cancer from benign lesions. Oncotarget.

[23] Liu L., Kawashima M., Sugimoto M., Sonomura K., Pu F., Li W., Takeda M., Goto T., Kawaguchi K., Sato T. (2023). Discovery of lipid profiles in plasma-derived extracellular vesicles as biomarkers for breast cancer diagnosis. Cancer Sci..

[24] Dorado E., Doria M.L., Nagelkerke A., McKenzie J.S., Maneta-Stavrakaki S., Whittaker T.E., Nicholson J.K., Coombes R.C., Stevens M.M., Takats Z. (2024). Extracellular vesicles as a promising source of lipid biomarkers for breast cancer detection in blood plasma. J. Extracell. Vesicles.

[25] Silva A.A.R., Cardoso M.R., Rezende L.M., Lin J.Q., Guimaraes F., Silva G.R.P., Murgu M., Priolli D.G., Eberlin M.N., Tata A. (2020). Multiplatform Investigation of Plasma and Tissue Lipid Signatures of Breast Cancer Using Mass Spectrometry Tools. Int. J. Mol. Sci..

[26] Assad D.X., Acevedo A.C., Mascarenhas E.C.P., Normando A.G.C., Pichon V., Chardin H., Guerra E.N.S., Combes A. (2020). Using an Untargeted Metabolomics Approach to Identify Salivary Metabolites in Women with Breast Cancer. Metabolites.

[27] Buentzel J., Klemp H.G., Kraetzner R., Schulz M., Dihazi G.H., Streit F., Bleckmann A., Menck K., Wlochowitz D., Binder C. (2021). Metabolomic Profiling of Blood-Derived Microvesicles in Breast Cancer Patients. Int. J. Mol. Sci..

[28] Eghlimi R., Shi X., Hrovat J., Xi B., Gu H. (2020). Triple Negative Breast Cancer Detection Using LC–MS/MS Lipidomic Profiling. J. Proteome Res..

[29] Fichtali K., Bititi A., Elghanmi A., Ghazi B. (2020). Serum Lipidomic Profiling in Breast Cancer to Identify Screening, Diagnostic, and Prognostic Biomarkers. BioRes. Open Access.

[30] Hilvo M., Denkert C., Lehtinen L., Müller B., Brockmöller S., Seppänen-Laakso T., Budczies J., Bucher E., Yetukuri L., Castillo S. (2011). Novel Theranostic Opportunities Offered by Characterization of Altered Membrane Lipid Metabolism in Breast Cancer Progression. Cancer Res..

[31] Ikarashi M., Tsuchida J., Nagahashi M., Takeuchi S., Moro K., Toshikawa C., Abe S., Ichikawa H., Shimada Y., Sakata J. (2021). Plasma Sphingosine-1-Phosphate Levels Are Associated with Progression of Estrogen Receptor-Positive Breast Cancer. Int. J. Mol. Sci..

[32] Iwano T., Yoshimura K., Inoue S., Odate T., Ogata K., Funatsu S., Tanihata H., Kondo T., Ichikawa D., Takeda S. (2020). Breast cancer diagnosis based on lipid profiling by probe electrospray ionization mass spectrometry. Br. J. Surg..

[33] Nishida-Aoki N., Izumi Y., Takeda H., Takahashi M., Ochiya T., Bamba T. (2020). Lipidomic Analysis of Cells and Extracellular Vesicles from High- and Low-Metastatic Triple-Negative Breast Cancer. Metabolites.

[34] Safari F., Kehelpannala C., Safarchi A., Batarseh A.M., Vafaee F. (2023). Biomarker Reproducibility Challenge: A Review of Non-Nucleotide Biomarker Discovery Protocols from Body Fluids in Breast Cancer Diagnosis. Cancers.

[35] Alba-Bernal A., Lavado-Valenzuela R., Domínguez-Recio M.E., Jiménez-Rodriguez B., Queipo-Ortuño M.I., Alba E., Comino-Méndez I. (2020). Challenges and achievements of liquid biopsy technologies employed in early breast cancer. EBioMedicine.

[36] Sun Y., Saito K., Saito Y. (2019). Lipid Profile Characterization and Lipoprotein Comparison of Extracellular Vesicles from Human Plasma and Serum. Metabolites.

[37] Ripley B.D. (2007). Pattern Recognition and Neural Networks.

[38] Marron J.S., Todd M.J., Ahn J. (2007). Distance-Weighted Discrimination. J. Am. Stat. Assoc..

[39] Kursa M.B., Rudnicki W.R. (2010). Feature Selection with the Boruta Package. J. Stat. Softw..

[40] Díaz-Beltrán L., González-Olmedo C., Luque-Caro N., Díaz C., Martín-Blázquez A., Fernández-Navarro M., Ortega-Granados A.L., Gálvez-Montosa F., Vicente F., del Palacio J.P. (2021). Human Plasma Metabolomics for Biomarker Discovery: Targeting the Molecular Subtypes in Breast Cancer. Cancers.

[41] Ide Y., Waki M., Hayasaka T., Nishio T., Morita Y., Tanaka H., Sasaki T., Koizumi K., Matsunuma R., Hosokawa Y. (2013). Human Breast Cancer Tissues Contain Abundant Phosphatidylcholine(36:1) with High Stearoyl-CoA Desaturase-1 Expression. PLoS ONE.

[42] Guo R., Chen Y., Borgard H., Jijiwa M., Nasu M., He M., Deng Y. (2020). The Function and Mechanism of Lipid Molecules and Their Roles in The Diagnosis and Prognosis of Breast Cancer. Molecules.

[43] Santoro A.L., Drummond R.D., Silva I.T., Ferreira S.S., Juliano L., Vendramini P.H., Lemos M.B.d.C., Eberlin M.N., Andrade V.P. (2020). In Situ DESI-MSI Lipidomic Profiles of Breast Cancer Molecular Subtypes and Precursor Lesions. Cancer Res..

[44] Gil-de-Gómez L., Balgoma D., Montero O. (2020). Lipidomic-Based Advances in Diagnosis and Modulation of Immune Response to Cancer. Metabolites.

[45] Tiwary S., Berzofsky J.A., Terabe M. (2019). Altered Lipid Tumor Environment and Its Potential Effects on NKT Cell Function in Tumor Immunity. Front. Immunol..

[46] Draijer L.G., Froon-Torenstra D., van Weeghel M., Vaz F.M., Bohte A.E., Holleboom A.G., Benninga M.A., Koot B.G. (2020). Lipidomics in Nonalcoholic Fatty Liver Disease. J. Pediatr. Gastroenterol. Nutr..

[47] Purroy F., Ois A., Jove M., Arque G., Sol J., Mauri-Capdevila G., Rodriguez-Campello A., Pamplona R., Portero M., Roquer J. (2023). Lipidomic signature of stroke recurrence after transient ischemic attack. Sci. Rep..

[48] Wortmann S.B., Vaz F.M., Gardeitchik T., Vissers L.E., Renkema G.H., Schuurs-Hoeijmakers J.H., Kulik W., Lammens M., Christin C., Kluijtmans L.A.J. (2012). Mutations in the phospholipid remodeling gene SERAC1 impair mitochondrial function and intracellular cholesterol trafficking and cause dystonia and deafness. Nat. Genet..

[49] Holčapek M., Cífková E., Lísa M., Jirásko R., Wolrab D., Hrnčiarová T. (2018). A Method of Diagnosing Pancreatic Cancer Based on Lipidomic Analysis of a Body Fluid. European Patent.

[50] Nam M., Seo S.S., Jung S., Jang S.Y., Lee J., Kwon M., Khan I., Ryu D.H., Kim M.K., Hwang G.-S. (2021). Comparable Plasma Lipid Changes in Patients with High-Grade Cervical Intraepithelial Neoplasia and Patients with Cervical Cancer. J. Proteome Res..

[51] Ottensmann L., Tabassum R., Ruotsalainen S.E., Gerl M.J., Klose C., Widén E., Gen F., Simons K., Ripatti S., Pirinen M. (2023). Genome-wide association analysis of plasma lipidome identifies 495 genetic associations. Nat. Commun..

[52] Seger C., Salzmann L. (2020). After another decade: LC–MS/MS became routine in clinical diagnostics. Clin. Biochem..

[53] Wang M., Wang C., Han X. (2022). Selection of internal standards for accurate quantification of complex lipid species in biological extracts by electrospray ionization mass spectrometry—What, how and why?. Mass Spectrom. Rev..

[54] Matyash V., Liebisch G., Kurzchalia T.V., Shevchenko A., Schwudke D. (2008). Lipid extraction by methyl-tert-butyl ether for high-throughput lipidomics. J. Lipid Res..

[55] Gachotte D., Adelfinskaya Y., Gilbert J., Kiyonami R., Peake D., Yokoi Y. Increased Depth and Confidence of Lipidome Analysis from Insect Tissues using Chromatography Based Methods with High-resolution Orbitrap MSn. Proceedings of the 66th ASMS Conference on Mass Spectrometry and Allied Topics.

[56] Yamada T., Uchikata T., Sakamoto S., Yokoi Y., Fukusaki E., Bamba T. (2013). Development of a lipid profiling system using reverse-phase liquid chromatography coupled to high-resolution mass spectrometry with rapid polarity switching and an automated lipid identification software. J. Chromatogr. A.

[57] Taguchi R., Ishikawa M. (2010). Precise and global identification of phospholipid molecular species by an Orbitrap mass spectrometer and automated search engine Lipid Search. J. Chromatogr. A.

[58] Peake D.A., Kiyonami R., Gachotte D., Reid G.E., Yokoi Y., Hühmer A. (2018). Software Utilizing Positive and Negative Ion MS2/MS3 HCD and CID Spectra for Improved MSn Lipid Identification.

[59] Peake D.A., Kiyonami R., Gachotte D., Reid G.E., Yokoi Y., Hühmer A. (2019). Increased Confidence of Insect Lipidome Annotation from High-Resolution Orbitrap LC/MSn Analysis and LipidSearch Software, Thermo Scientific Application Note 72942. https://assets.thermofisher.cn/TFS-Assets/CMD/Application-Notes/an-72942-lc-ms-insect-lipidome-an72942-en.pdf.

[60] Plubell D.L., Wilmarth P.A., Zhao Y., Fenton A.M., Minnier J., Reddy A.P., Klimek J., Yang X., David L.L., Pamir N. (2017). Extended Multiplexing of Tandem Mass Tags (TMT) Labeling Reveals Age and High Fat Diet Specific Proteome Changes in Mouse Epididymal Adipose Tissue*. Mol. Cell. Proteom..

[61] Kuhn M. (2008). Building Predictive Models in R Using the caret Package. J. Stat. Softw..

