# Supplementary material for: Discovery of Plasma Lipids as Potential Biomarkers Distinguishing Breast Cancer Patients from Healthy Controls
Source: Int J Mol Sci. 2024 Oct 28;25(21):11559. doi: 10.3390/ijms252111559 (PMC11546708; doi:10.3390/ijms252111559)
Supplement: Supplementary file 1 [file ijms-25-11559-s001.zip › Supp figures and legends.pdf]

Supplementary Data

Discovery of Plasma Lipids as Potential Biomarkers Distinguishing Breast Cancer Patients from Healthy Controls

Desmond Li 1, Kerry Heffernan 1, Forrest C. Koch 2, David A. Peake 1, Dana Pascovici 3, Mark David 1, Cheka Kehelpannala 1, G. Bruce Mann 4, David Speakman 5,6, John Hurrell 1, Simon Preston 1,*, Fatemeh Vafaee 2,7 and Amani Batarseh 1

1 BCAL Diagnostics Ltd., Sydney, NSW 2000, Australia
2 OmniOmics.ai Pty Ltd., Pagewood, NSW 2035, Australia
3 InsightStats, Croydon Park, NSW 2133, Australia
4 Department of Surgery, The Royal Melbourne Hospital, Parkville, VIC 3050, Australia
5 The Peter MacCallum Cancer Centre, Sir Peter MacCallum Department of Oncology, University of Melbourne, Melbourne, VIC 3010, Australia
6 BreastScreen Victoria, Carlton, VIC 3053, Australia
7 School of Biotechnology and Biomolecular Sciences, University of New South Wales (UNSW), Sydney, NSW 2052, Australia

Supplementary Figure S1

A

	Cohort 1		Cohort 2		Cohort 3		Combined	
Age	Control	Cancer	Control	Cancer	Control	Cancer	Control	Cancer
21-30	0	0	0	0	2	1	2	1
31-40	3	4	14	11	11	6	28	21
41-50	15	15	25	25	24	28	64	68
51-60	23	29	40	36	22	24	85	89
61-70	31	37	17	17	30	33	78	87
71-80	19	10	4	10	10	6	33	26
80+	9	4	0	1	1	1	10	6
Totals	100	99	100	100	100	99	300	298
Totals	199		200		199		598	

B

		Subjects			
Disease	Stage	Cohort 1	Cohort 2	Cohort 3	Combined
Control	N/A	100	100	100	300
DCIS	0	0	0	51	51
IDC	I	50	50	0	100
IDC	II	49	50	0	99
ILC	I	0	0	20	20
ILC	II	0	0	28	28
Totals	N/A	199	200	199	598

C

			Age		BMI		Smoking history		
	Disease	Subjects	Mean (SD)	p-value	Mean (SD)	p-value	Yes	No	p-value
Cohort 1	Control	100	63.3 (12.4)	0.057	28.0 (4.4)	0.600	19	81	0.357
	Cancer	99	60.2 (10.6)		28.4 (4.4)		14	85	
Cohort 2	Control	100	52.8 (10.3)	0.253	26.8 (4.3)	0.675	17	83	0.054
	Cancer	100	54.6 (11.4)		27.0 (4.8)		8	92	
Cohort 3	Control	100	55.3 (12.3)	0.581	27.4 (4.8)	0.709	11	89	0.003
	Cancer	99	56.2 (11.2)		27.2 (4.4)		1	98	
Combined	Control	300	57.1 (12.5)	0.872	27.4 (4.5)	0.757	47	253	0.003
	Cancer	298	57.0 (11.3)		27.5 (4.6)		23	275	

D

LID	Annotation	*m/z*	Cohort 1	Cohort 2
033	LPC 14:0	468.3085	No	Yes
084	PC 32:1	732.5538	Yes	Yes
102	PC 36:2	786.6007	Yes	No
123	PC 38:5	808.5851	Yes	Yes
124	PC 38:5	808.5851	Yes	Yes
125	PC 38:5	808.5851	Yes	Twin m/z
174	PE 34:1	718.5381	Yes	Yes
223	PI 34:1	835.5342	Yes	No
245	PS 38:4	810.5291	Yes	No
276	SM d36:2	729.5905	Yes	Yes
281	SM d38:4	753.5905	Yes	Yes
313	SM d44:4	837.6844	No	No
454	TG O-52:3	860.8066	Yes	Yes
378	TG 53:4	886.7858	Yes	Yes
383	TG 54:4	900.8015	Yes	No
384	TG 54:5	898.7858	Twin m/z	No
385	TG 54:5	898.7858	Yes	No
386	TG 54:6	879.7436	Twin m/z	Yes
388	TG 54:6	896.7702	Yes	Twin m/z
387	TG 54:6	896.7702	Twin m/z	Twin m/z
400	TG 56:1	934.8797	Yes	Yes
423	TG 58:2	960.8954	Yes	Yes
424	TG 58:3	958.8797	Yes	Yes

Supplementary Figure S1. Sample donor demographics and lipid species annotation for the EV logistic regression discovery dataset (n=598). (A) Number of donor subjects in each cohort, disease status and age at time of sample collection. (B) Number of donor subjects by breast cancer subtype in each cohort. (C) Age, BMI and smoking history of donor subjects in each cohort. Age and BMI were compared using a student's *t*-test and

smoking history using a chi-squared test. (D) List of 23-lipid species identified in EVs that discriminated between healthy controls and individuals with breast cancer. The lipids that were significantly increased, or decreased, in breast cancer samples in cohort 1 and cohort 2 are indicated. LID, lipid identifier; Annotation, sum composition; *m*/*z*, mass to charge ratio; SD, standard deviation.

Supplementary Figure S2

A

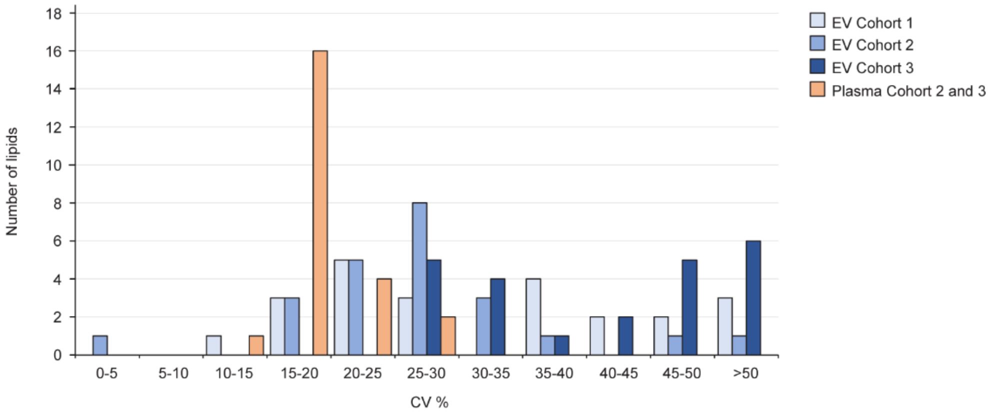

B

	Cohort 2		Cohort 3		Combined	
Age	Control	Cancer	Control	Cancer	Control	Cancer
21-30	0	0	0	1	0	1
31-40	5	5	5	6	10	11
41-50	9	7	9	28	18	35
51-60	22	16	15	24	37	40
61-70	15	13	13	33	28	46
71-80	4	10	7	6	11	16
80+	0	1	1	1	1	2
Totals	55	52	50	99	105	151
Totals	107		149		256	

C

		Subjects		
Disease	Stage	Cohort 2	Cohort 3	Combined
Control	N/A	55	50	105
DCIS	0	0	51	51
IDC	I	23	0	23
IDC	II	29	0	29
ILC	I	0	20	20
ILC	II	0	28	28
Totals	N/A	107	149	256

D

			Age		BMI		Smoking history		
	Disease	Subjects	Mean (SD)	p-value	Mean (SD)	p-value	Yes	No	p-value
Cohort 2	Control	55	56.9 (10.5)	0.381	27.8 (4.3)	0.504	5	50	0.794
	Cancer	52	58.9 (12.2)		28.4 (4.8)		4	48	
Cohort 3	Control	50	57.5 (11.8)	0.510	27.1 (5.1)	0.937	6	44	0.003
	Cancer	99	56.2 (11.2)		27.2 (4.4)		1	98	
Combined	Control	105	57.2 (11.1)	0.952	27.5 (4.7)	0.841	11	94	0.020
	Cancer	151	57.1 (11.6)		27.6 (4.6)		5	146	

E

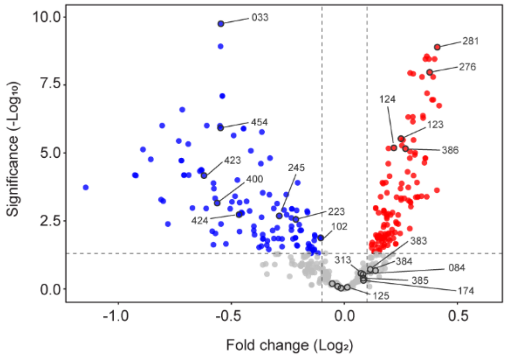

Supplementary Figure S2. Lipid acquisition reproducibility and sample donor demographics for the plasma logistic regression discovery dataset (n=256). (A) The coefficients of variation (CV%) distributions for the 23-lipids in QC samples. Different QC

sample batches were used for the indicated groups. (B) Number of donor subjects in each cohort, disease status and age at time of sample collection. (C) Number of donor subjects by breast cancer subtype in each cohort. (D) Age, BMI and smoking history of donor subjects in each cohort. Age and BMI were compared using a student's *t*-test and smoking history using a chi-squared test. (E) Volcano plot of lipid profile identified in plasma from breast cancer subjects compared to control donors. The fold-change in relative lipid expression is shown. Lipid species that were significantly decreased (blue) or increased (red) in breast cancer samples are indicated and lipids in the 23-lipid panel are annotated. SD, standard deviation.

Supplementary Figure S3

A

LID	Annotation	Adduct	Rt, min	m/z	MW
238	PI 38:6	M-H	6.223	881.5186	882.5258
098	PC 36:0	M+H	12.46	790.6320	789.6248
244	PS 38:4	M-H	8.806	810.5291	811.5363
208	PE P-34:2	M+H	9.427	700.5276	699.5203
204	PE O-40:6	M-H	10.941	776.5600	777.5672
231	PI 36:4	M-H	6.375	857.5186	858.5258
221	PG 36:1	M-H	10.445	775.5495	776.5567
212	PE P-36:5	M+H	8.11	722.5119	721.5046
243	PS 36:1	M-H	10.286	788.5447	789.552
273	SM d35:1	M+H	8.426	717.5905	716.5832
454	TG O-52:3	M+NH4	16.938	860.8066	842.7727
033	LPC 14:0	M+H	1.973	468.3085	467.3012
089	PC 34:0	M+H	11.078	762.6007	761.5935
418	TG 58:1	M+NH4	17.521	962.9110	944.8772
209	PE P-36:2	M+H	11.174	728.5589	727.5516
247	PS 40:7	M-H	8.792	832.5134	833.5207
284	SM d39:2	M+H	10.258	771.6375	770.6302
296	SM d41:2	M+H	11.798	799.6688	798.6615
373	TG 53:0	M+NH4	17.397	894.8484	876.8146
423	TG 58:2	M+NH4	17.523	960.8954	942.8615

B

Model	Description	Acc	F1	TPR	TNR	PPV
**dwdPoly**	Distance Weighted Discrimination (Polynomial Kernel)	0.863	0.884	0.882	0.836	0.886
**avNNet**	Neural Networks Using Model Averaging	0.861	0.884	0.899	0.806	0.870
**ensemble**	Majority Vote	0.861	0.885	0.904	0.802	0.867
**svmRadial**	Support Vector Machine (Radial Kernel)	0.856	0.877	0.873	0.830	0.881
**svmRadialSigma**	Support Vector Machine (Radial Sigma Kernel)	0.853	0.876	0.874	0.823	0.877
**gaussprRadial**	Gaussian Process (Radial Kernel)	0.845	0.870	0.875	0.803	0.865
**vglmAdjCat**	Adjacent Categories Model	0.844	0.870	0.883	0.787	0.857
**dwdRadial**	Distance Weighted Discrimination (Radial Kernel)	0.843	0.869	0.878	0.793	0.860
**ranger**	Random Forests	0.836	0.864	0.884	0.766	0.845
**vglmCumulative**	Cumulative Probability Model	0.836	0.864	0.883	0.769	0.846
**gbm**	Generalised Boosted Models	0.828	0.856	0.867	0.773	0.846
**xgbTree**	eXtreme Gradient Boosted Trees	0.823	0.853	0.866	0.762	0.840
**mlp**	Multi-Layer Perceptron	0.818	0.849	0.864	0.753	0.834
**knn**	K-Nearest Neighbours	0.814	0.838	0.813	0.815	0.864
**CSimca**	Soft Independent Modelling of Class Analogy	0.812	0.854	0.933	0.638	0.788
**ownn**	Optimal Weighted Nearest Neighbour	0.812	0.837	0.815	0.809	0.860
**svmLinearWeights2**	Support Vector Machine (Linear Kernel)	0.798	0.840	0.896	0.657	0.790
**deepboost**	Deep Boosting	0.796	0.835	0.876	0.681	0.798
**mlpWeightDecaML**	Multi-Layer Perceptron with Weighted Decay	0.789	0.825	0.839	0.717	0.811

Supplementary Figure S3. Breast cancer prediction models using EV-derived lipids as features. (A) Table indicating the lipid species in the EV20 panel, with corresponding LID, annotation (sum composition), adduct, retention time (Rt, in minutes), mass/charge (*m/z*) and molecular weight (MW). (B) Average performance measures of 19 classifiers, including the ensemble model across 2000 LGOCV (20% test, 80% train) where each model uses the EV20 panel as its predictive variables. Acc, accuracy; F1, F1-

score, TPR, true positive rate (sensitivity); TNR, true negative rate (specificity); PPV, positive predictive value.

Supplementary Figure S4

A

LID	Annotation	Adduct	Rt (min)	m/z	MW
202	PE O-38:6	M-H	9.79	748.5287	749.5359
102	PC 36:2	M+H	9.90	786.6007	785.5935
100	PC 36:2	M+H	9.90	786.6007	785.5935
105	PC 36:4	M+H	7.17	782.5694	781.5622
033	LPC 14:0	M+H	1.97	468.3085	467.3012
208	PE P-34:2	M+H	9.43	700.5276	699.5203
209	PE P-36:2	M+H	11.17	728.5589	727.5516
244	PS 38:4	M-H	8.81	810.5291	811.5363
204	PE O-40:6	M-H	10.94	776.5600	777.5672
243	PS 36:1	M-H	10.29	788.5447	789.5520
231	PI 36:4	M-H	6.38	857.5186	858.5258
035	LPC 16:0	M+H	2.84	496.3398	495.3325
225	PI 36:1	M-H	10.03	863.5655	864.5728
221	PG 36:1	M-H	10.45	775.5495	776.5567
211	PE P-36:3	M+H	9.46	726.5432	725.5359
325	TG 44:2	M+NH4	15.05	764.6763	746.6424
454	TG O-52:3	M+NH4	16.94	860.8066	842.7727
324	TG 44:2	M+NH4	15.05	764.6763	746.6424
228	PI 36:3	M-H	7.37	859.5342	860.5415
212	PE P-36:5	M+H	8.11	722.5119	721.5046

B

Model	Description	Acc	F1	TPR	TNR	PPV
**ensemble**	Majority Vote	0.861	0.886	0.913	0.786	0.860
**avNNet**	Neural Networks Using Model Averaging	0.857	0.880	0.888	0.814	0.873
**dwdPoly**	Distance Weighted Discrimination (Polynomial Kernel)	0.854	0.881	0.915	0.765	0.849
**knn**	K-Nearest Neighbours	0.854	0.878	0.888	0.805	0.868
**gaussprRadial**	Gaussian Process (Radial Kernel)	0.851	0.876	0.889	0.796	0.863
**dwdRadial**	Distance Weighted Discrimination (Radial Kernel)	0.850	0.876	0.894	0.787	0.858
**ranger**	Random Forests	0.848	0.874	0.889	0.789	0.859
**svmRadialSigma**	Support Vector Machine (Radial Sigma Kernel)	0.848	0.877	0.914	0.752	0.842
**ownn**	Optimal Weighted Nearest Neighbour	0.846	0.871	0.880	0.796	0.862
**svmLinearWeights**	Support Vector Machine (Linear Kernel)	0.842	0.871	0.903	0.753	0.841
**mlpWeightDecayML**	Multi-Layer Perceptron with Weighted Decay	0.841	0.866	0.874	0.794	0.859
**svmRadial**	Support Vector Machine (Radial Kernel)	0.840	0.869	0.899	0.756	0.842
**gbm**	Generalised Boosted Models	0.839	0.865	0.878	0.782	0.853
**xgbTree**	eXtreme Gradient Boosted Trees	0.838	0.865	0.876	0.784	0.854
**mlp**	Multi-Layer Perceptron	0.836	0.861	0.863	0.798	0.860
**vglmAdjCat**	Adjacent Categories Model	0.822	0.849	0.851	0.779	0.848
**CSimca**	Soft Independent Modelling of Class Analogy	0.820	0.851	0.868	0.750	0.834
**vglmCumulative**	Cumulative Probability Model	0.820	0.848	0.848	0.780	0.847
**deepboost**	Deep Boosting	0.819	0.849	0.858	0.764	0.840

Supplementary Figure S4. Breast cancer prediction models using plasma-derived lipids as features. (A) Table indicating the lipid species in the P20 panel, with corresponding LID, annotation (sum composition), adduct, retention time (Rt, in minutes), mass/charge (*m/z*) and molecular weight (MW). (B) Average performance measures of 19 classifiers, including the ensemble model across 2000 LGOCV (20% test, 80% train) where each model uses the P20 panel as its predictive variables. Acc, accuracy; F1, F1-score, TPR, true positive rate (sensitivity); TNR, true negative rate (specificity); PPV, positive predictive value.

Supplementary Figure S5

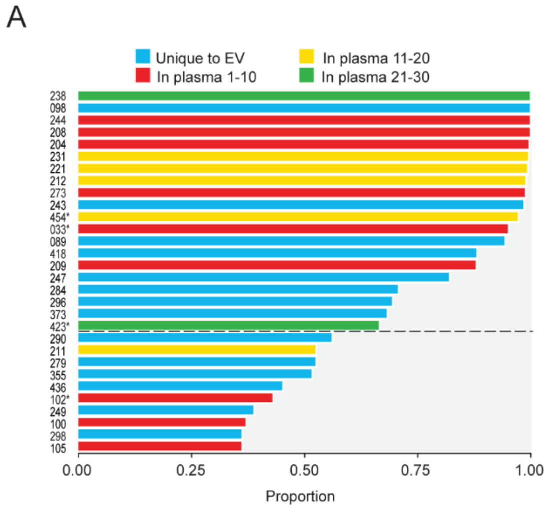

B

LID	Annotation	EV23	EV20	P20
387	TG 54:6	■		
388	TG 54:6	■		
385	TG 54:5	■		
383	TG 54:4	■		
223	PI 34:1	■		
384	TG 54:4	■		
245	PS 38:4	■		
276	SM d36:2	■		
378	TG 53:4	■		
123	PC 38:5	■		
124	PC 38:5	■		
125	PC 38:5	■		
084	PC 32:1	■		
174	PE 34:1	■		
281	SM d38:4	■		
400	TG 56:1	■		
424	TG 58:3	■		
313	SM d44:4	■		
386	TG 54:6	■		
273	SM d35:1		■	
089	PC 34:0		■	
098	PC 36:0		■	
238	PI 38:6		■	
247	PS 40:7		■	
284	SM d39:2		■	
373	TG 53:0		■	
418	TG 58:1		■	
296	SM d41:2		■	
228	PI 36:3			■
225	PI 36:1			■
211	PE P-36:3			■
035	LPC 16:0			■
105	PC 36:4			■
100	PC 36:2			■
202	PE O-38:6			■
324	TG 44:2			■
325	TG 44:2			■

□ Not present
■ Present

Supplementary Figure S5. Comparison of the lipids that were consistently selected as being important in both plasma and EVs using the machine learning discovery pipeline. (A) Top 30 lipids that were consistently selected as being important by the Boruta algorithm across all EV runs are shown. Blue bars indicate lipids that were unique to the EV analysis. Red, yellow and green bars indicate lipids that were also identified in the top 30 lipids in the plasma analysis. Lipids identified in the EV23 panel are indicated with an asterisk (*) next to the LID. The cutoff between the top 20 and the remaining 10 lipids is indicated with a dotted line. (B) Table indicating the lipid species that were only identified in their respective panels and not in others. Number of lipid species correspond to the numbers in the non-overlapping sections of the Venn diagram in figure 5D.
